# Assessing Readability of Skin Cancer Screening Resources: A Comparison of Online Websites and ChatGPT Responses

**DOI:** 10.1007/s13187-025-02683-2

**Published:** 2025-07-01

**Authors:** Elissa Goorman, Sukul Mittal, Jennifer N. Choi

**Affiliations:** 1 Department of Dermatology, Northwestern University Feinberg School of Medicine, Chicago, IL USA; 2 Jacobs School of Medicine and Biomedical Sciences, Buffalo, NY USA; 3 University of Texas Southwestern School of Medicine, Dallas, TX USA

**Keywords:** Skin cancer screening, Readability, Online health information, ChatGPT, Patient education, Health literacy, Artificial intelligence

## Abstract

Effective communication is essential for promoting appropriate skin cancer screening for the public. This study compares the readability of online resources and ChatGPT-generated responses related to the topic of skin cancer screening. We analyzed 60 websites and responses to five questions from ChatGPT-4.0 using five readability metrics: the Flesch-Kincaid Reading Ease, Flesch-Kincaid Grade Level, SMOG Index, Gunning Fog Index, and Coleman-Liau Index. Results showed that both websites and ChatGPT responses exceeded the recommended sixth grade reading level for health-related information. No significant differences were found between the readability for university-hosted versus non-university-hosted websites. However, across all readability metrics, ChatGPT responses were significantly more difficult to read. These findings highlight the need to enhance the accessibility of health information by aligning content with recommended literacy levels. Future efforts should focus on developing patient-centered, publicly accessible materials and refining AI-generated content to improve public understanding and encourage proactive engagement in skin cancer screenings.

## Introduction

Skin cancer is a significant public health concern, but the lack of consensus on screening recommendations creates uncertainty for both physicians and patients [1]. The US Preventive Services Task Force (USPSTF) has stated that the evidence is insufficient to determine the balance of benefits and harms for routine visual skin examinations by clinicians in asymptomatic individuals, leaving patients unsure about when to seek screenings [1, 2].

Frequently, patients turn to online resources to fill these gaps in guidance. Websites are often the first stop for individuals seeking health information, as they offer immediate access to educational content [3]. However, prior studies have shown that the readability of online patient education materials is often too complex for the general public, limiting their utility [4, 5]. Common readability tests include the Flesch-Kincaid Reading Ease (FRE), Flesch-Kincaid Grade Level (FKGL), Gunning Fog Index (GFI), Coleman-Liau Index (CLI), and Simple Measure of Gobbledygook (SMOG) Index, with each measuring readability using different formulas. The FKGL and FRE use average words per sentence and syllables per word, while the GFI bases its calculation on sentence length, as well as the number of words containing three or more syllables [4]. CLI does not analyze the content of words, but simply their length in characters, and the SMOG Index counts words with three or more syllables in a subset of text from a written sample [4]. Past research has demonstrated the FKGL is the most commonly used index, but can underestimate reading difficulty, while the SMOG Index is recommended as the gold standard for consumer-oriented healthcare materials [6].

In addition to websites, the rise of ChatGPT presents a new tool for accessing health information. Unlike static web pages, ChatGPT offers interactive, conversational responses tailored to individual queries, potentially improving accessibility [7]. However, with its growing popularity, it is essential to evaluate the readability and understandability of ChatGPT’s responses alongside traditional online sources to ensure that patients receive clear, understandable information, regardless of where they search. This study aims to assess and compare the readability of both websites and ChatGPT-generated responses related to skin cancer screening, offering insights into the accessibility of these resources and identifying potential areas for improvement.

## Methods

This cross-sectional study assessed the readability of online resources and ChatGPT-generated responses regarding skin cancer screening. A search was conducted on October 26, 2024, using the Chrome browser in Incognito mode, with cache cleared to avoid personalized results. The term “skin cancer screening” was entered, and the first 100 websites were reviewed. Only English-language articles were included. URLs were excluded if they led to splash pages, contained irrelevant information, or were news articles, clinical trial databases, genome data, peer-reviewed literature, books, or provider-focused materials. Websites were categorized as university-hosted or non-university-hosted for subgroup analysis.

Five standardized questions on skin cancer screening were submitted three times each to ChatGPT-4.0 Mini in separate Incognito sessions to minimize response variability. We asked ChatGPT—(1) What should I expect at a skin cancer screening, (2) When should I get my skin cancer screening, (3) Where should I get my skin cancer screening, (4) Who should do my skin cancer screening, and (5) How do I do a skin cancer screening at home? The responses were analyzed alongside website content using Readable.io, a validated tool for automating readability assessments [8]. The metrics applied included the FRE, FKGL, GFI, CLI, and SMOG Index. An FRE score below 60 was considered moderately difficult, while a grade level above 10 indicated high reading difficulty.

Data analysis was conducted using SPSS version 29.0. Continuous variables were described using means and standard deviations. Student’s *t*-tests were used to compare university-hosted and non-university-hosted websites, as well as to compare all websites with ChatGPT responses. Statistical significance was determined at a threshold of *p* < 0.05.

## Results

Of the 100 websites reviewed, 60 met the inclusion criteria.

### Readability of Websites

The FRE score for websites averaged 58.47 (SD = 10.69), indicating moderately difficult content suitable for late high school readers. The FKGL averaged 8.16 (SD = 1.82), corresponding to middle school level. Other metrics confirmed higher reading difficulty, with the SMOG Index at 10.72 (SD = 1.46) and the CLI at 10.16 (SD = 2.00), placing most materials in the high school to college range (Table 1).

**Table 1:** Overview of readability and understandability scores used for all websites, university-hosted websites, non-university-hosted websites, and ChatGPT responses

Responses	Average scores (SD)	Corresponding reading grade level	Corresponding readability difficulty
**All websites (*n* = 60)**			
Flesch-Kincaid Reading Ease	58.47 (10.69)	Late high school	Moderately difficult
Flesch-Kincaid Grade Level	8.16 (1.82)	Middle school	Average
SMOG Index	10.72 (1.46)	Late high school	Difficult
Gunning Fog Index	9.56 (2.11)	Early high school	Moderately difficult
Coleman-Liau Index	10.16 (2.00)	Late high school	Difficult
**University-hosted websites (*n* = 16)**			
Flesch-Kincaid Reading Ease	58.8 (8.69)	Late high school	Moderately difficult
Flesch-Kincaid Grade Level	7.79 (1.62)	Middle school	Average
SMOG Index	10.51 (1.24)	Late high school	Difficult
Gunning Fog Index	9.07 (1.83)	Early high school	Moderately difficult
Coleman-Liau Index	10.31 (1.77)	Late high school	Difficult
**Non-university-hosted websites (*n* = 44)**			
Flesch-Kincaid Reading Ease	58.35 (11.41)	Late high school	Moderately difficult
Flesch-Kincaid Grade Level	8.29 (1.88)	Middle school	Average
SMOG Index	10.79 (1.54)	Late high school	Difficult
Gunning Fog Index	9.74 (2.19)	Early high school	Moderately difficult
Coleman-Liau Index	10.11 (2.10)	Late high school	Difficult
**ChatGPT responses**			
Flesch-Kincaid Reading Ease	46.27 (11.44)	College	Difficult
Flesch-Kincaid Grade Level	11.05 (2.66)	Early high school	Difficult
SMOG Index	13.33 (2.29)	College	Difficult
Gunning Fog Index	13.39 (3.74)	College	Difficult
Coleman-Liau Index	11.79 (1.92)	Late high school	Difficult

### Distribution of Readability Scores

As shown in Table 2, 47% of websites had FRE scores in the difficult range (0–59), while 53% scored in the average range (60–79). None was categorized as easy (80–100). On the FKGL, 88% of websites were written above a 6th-grade level, with two websites also exceeding the 10th-grade level. The SMOG and Gunning Fog indices indicated that most content required at least a high school reading level.

**Table 2:** Distribution of readability scores by category

Readability scores	No. of websites (*n* = 60)
**Flesch-Kincaid Reading Ease**	
Easy (80–100)	0
Average (60–79)	32 (53%)
Difficult (0–59)	28 (47%)
**Flesch-Kincaid Grade Level**	
Up to grade 6	7 (12%)
Grade 7–10	51 (85%)
Beyond grade 10	2 (3%)
**SMOG Index**	
Up to grade 6	0
Grade 7–10	20 (33%)
Beyond grade 10	40 (67%)
**Gunning Fog Index**	
Up to grade 6	17 (28%)
Grade 7–10	25 (42%)
Beyond grade 10	18 (30%)
**Coleman-Liau Index**	
Up to grade 6	0
Grade 7–10	21 (35%)
Beyond grade 10	39 (65%)

### University vs. Non-University Websites

Sixteen (26.7%) of all websites were university-hosted websites, while 44 (73.3%) were not. There were no significant differences between university and non-university websites across all readability metrics. The average FRE score was 58.8 (SD = 8.69) for university-hosted websites and 58.35 (SD = 11.41) for non-university-hosted websites (*p* = 0.88). The FKGL was 7.79 (SD = 1.62) for university-hosted websites and 8.29 (SD = 1.88) for non-university websites (*p* = 0.35). Other metrics, including the SMOG and Gunning Fog indices, showed similar patterns (Table 1).

### ChatGPT Responses

The average FRE score was 46.27 (SD = 11.44), corresponding to difficult content suitable for college readers. The FKGL, SMOG, Gunning Fog, and Coleman-Liau indices repeat this pattern, with content presenting at a difficult readability level (Table 1).

### Comparison Between Websites and ChatGPT Responses

Table 3 demonstrates that ChatGPT responses were consistently more challenging to read than the websites across all readability metrics. The FKGL score for ChatGPT responses was significantly higher than that of websites (11.05 vs. 8.16; *p* < 0.001). Similarly, the SMOG index for ChatGPT was elevated compared to websites (13.33 vs. 10.72; *p* < 0.001), as were the Gunning Fog scores (13.39 vs. 9.56; *p* < 0.0001). ChatGPT also had a significantly higher CLI score than websites (11.79 vs. 10.16; *p* = 0.0004). Moreover, the FRE score for ChatGPT was significantly lower, indicating poorer readability (46.27 vs. 58.47; *p* = 0.00021).

**Table 3:** Readability and understandability scores for all websites versus ChatGPT-generated responses

Tool	All websites (mean (SD))	ChatGPT (mean (SD))	*p*-value
Flesch-Kincaid Reading Ease	58.47 (10.69)	46.27 (11.44)	0.00021*
Flesch-Kincaid Grade Level	8.16 (1.82)	11.05 (2.66)	< 0.001*
SMOG Index	10.72 (1.46)	13.33 (2.29)	< 0.001*
Gunning Fog Index	9.56 (2.11)	13.39 (3.74)	< 0.001*
Coleman-Liau Index	10.16 (2.00)	11.79 (1.92)	0.0004*

## Discussion

The National Institutes of Health (NIH) and American Medical Association (AMA) readability guidelines recommend that educational materials be written at a sixth grade reading level or lower [9]. However, our analysis found that most websites were written at a late high school or college level, as indicated by the Gunning Fog and SMOG indices. Regarding the variability in some indices’ results, we may assign stronger weight to the results of the SMOG Index, as this is the gold standard of readability indices for medical content [6]. The findings are consistent with prior research demonstrating that online health materials are often too complex for individuals with average literacy levels [4, 5, 10]. For example, Basch et al. (2018) found that over 90% of skin cancer–related websites failed to meet recommended readability standards, a trend that our data also reflects [4].

The lack of significant readability differences between university and non-university websites aligns with previous studies that have shown similar challenges across educational and commercial sources [10]. Although it could be expected that university websites accommodate patients from diverse educational backgrounds, our findings highlight the widespread difficulty of producing easily understandable health materials, regardless of the source.

Similarly, ChatGPT responses, while designed to provide accessible information, required an even higher reading level, further emphasizing the challenges patients may face in accessing understandable health information. ChatGPT’s responses required college-level reading proficiency, raising concerns about usability for patient populations with diverse educational backgrounds. This suggests that while AI tools present new opportunities for health communication, they require further refinement to match the literacy needs of the general population.

The importance of accessible information in skin cancer screening cannot be overstated. Early detection through screening plays a critical role in reducing skin cancer morbidity and mortality [11]. However, if patients are unable to comprehend educational materials due to high readability demands, they may miss essential information about risk factors, self-examination techniques, and the need for timely screenings. This barrier can result in delayed diagnosis and poorer health outcomes, particularly among populations with lower health literacy. Healthcare providers and content creators should prioritize developing educational materials that adhere to NIH and AMA readability guidelines. Future research should explore the integration of readability tools into the creation of patient education content, as well as patient-centered approaches to content development to ensure accessibility across literacy levels. Improving the readability of online health content is also essential for empowering patients to make informed decisions about their health [12].

This study has several limitations. The use of only the first 100 search results and English-language content may limit generalizability. Additionally, the cross-sectional design captures readability at a single point in time, while website content may change. Future studies should examine patient comprehension directly through surveys or interviews to assess the real-world impact of readability on patient outcomes.

## Conclusion

This study reveals that both traditional websites and AI-generated responses, such as those from ChatGPT, present readability challenges that could hinder patient comprehension of skin cancer screening information. With both sources exceeding the recommended literacy level, there is a clear need to refine health communication strategies. Future efforts should focus on creating patient-centered content that is not only informative but also accessible across varying literacy levels.

## References

[1] Force UPST (2016) Screening for skin cancer: US Preventive Services Task Force recommendation statement. JAMA 316(4):429–435 27458948 10.1001/jama.2016.8465

[2] Force UPST (2023) Screening for skin cancer: US Preventive Services Task Force recommendation statement. JAMA 329(15):1290–1295 37071089 10.1001/jama.2023.4342

[3] Chu JT et al (2017) How, when and why people seek health information online: qualitative study in Hong Kong. Interact J Med Res 6(2):e24 29233802 10.2196/ijmr.7000 PMC5743920

[4] Basch CH et al (2018) Readability of online material related to skin cancer. Public Health 163:137–140 30149263 10.1016/j.puhe.2018.07.009

[5] MacLean SA et al (2018) Readability of information on colonoscopy preparation on the internet. Health Promot Perspect 8(2):167–170 29744314 10.15171/hpp.2018.22 PMC5935822

[6] Fitzsimmons PR et al (2010) A readability assessment of online Parkinson’s disease information. J R Coll Physicians Edinb 40(4):292–296 21132132 10.4997/JRCPE.2010.401

[7] OpenAI. ChatGPT: optimizing language models for dialogue. 2022; Available from: https://openai.com/blog/chatgpt/

[8] Health NIO. 2018 How to write easy-to_read materials. Last accessed on

[9] Weiss BD (2003) Health Lit Am Med Assoc 253:358

[10] Kaundinya T, El-Behaedi S, Choi JN (2023) Readability of online patient education materials for graft-versus-host disease. J Cancer Educ 38(4):1363–1366 36795293 10.1007/s13187-023-02271-2 PMC9933791

[11] Henrikson NB et al (2023) Skin cancer screening: updated evidence report and systematic review for the US Preventive Services Task Force. JAMA 329(15):1296–1307 37071090 10.1001/jama.2023.3262 PMC13178561

[12] Fitzpatrick PJ (2023) Improving health literacy using the power of digital communications to achieve better health outcomes for patients and practitioners. Front Digit Health 5:1264780 38046643 10.3389/fdgth.2023.1264780 PMC10693297

